# Point-of-Care Diagnostic System for Viable *Salmonella* Species via Improved Propidium Monoazide and Recombinase Polymerase Amplification Based Nucleic Acid Lateral Flow

**DOI:** 10.3390/diagnostics14080831

**Published:** 2024-04-17

**Authors:** So-Young Lee, Se-Wook Oh

**Affiliations:** Department of Food and Nutrition, Kookmin University, Seoul 136-702, Republic of Korea; leesoyoung0423@kookmin.ac.kr

**Keywords:** point-of-care diagnosis, *Salmonella*, RPA, PMAxx, nucleic acid lateral flow

## Abstract

*Salmonella* species are prominent foodborne microbial pathogens transmitted through contaminated food or water and pose a significant threat to human health. Accurate and rapid point-of-care (POC) diagnosis is gaining attention in effectively preventing outbreaks of foodborne disease. However, the presence of dead bacteria can interfere with an accurate diagnosis, necessitating the development of methods for the rapid, simple, and efficient detection of viable bacteria only. Herein, we used an improved propidium monoazide (PMAxx) to develop a nucleic acid lateral flow (NALF) assay based on recombinase polymerase amplification (RPA) to differentiate viable *Salmonella* Typhimurium. We selected an RPA primer set targeting the *invA* gene and designed a probe for NALF. RPA-based NALF was optimized for temperature (30–43 °C), time (1–25 min), and endonuclease IV concentration (0.025–0.15 unit/µL). PMAxx successfully eliminated false-positive results from dead *S*. Typhimurium, enabling the accurate detection of viable *S*. Typhimurium with a detection limit of 1.11 × 10^2^ CFU/mL in pure culture. The developed method was evaluated with spiked raw chicken breast and milk with analysis completed within 25 min at 39 °C. This study has potential as a tool for the POC diagnostics of viable foodborne pathogens with high specificity, sensitivity, rapidity, and cost-effectiveness.

## 1. Introduction

*Salmonella* species are primarily found in animals and the environment and are a major cause of human food-borne infection [1]. *Salmonella* species contamination frequently occurs during food distribution processes and is associated with foodborne illnesses and gastrointestinal diseases [2,3]. In particular, the consumption of poultry, milk, and fresh food is closely linked to *Salmonella* species contamination, and rapid and accurate pathogen detection is therefore critical for food safety and health [4]. Salmonellosis affects approximately 93 million people worldwide and causes symptoms such as diarrhea, fever, abdominal pain, vomiting, and nausea, which can be fatal to immunocompromised patients [5].

Therefore, the rapid detection of *Salmonella* species is crucial for public health and industrial purposes [6]. The traditional culture-based methods used to detect foodborne pathogens are time-consuming, which is often compounded by the necessity for additional biochemical testing [7]. Real-time PCR is widely regarded as the standard approach for rapidly detecting pathogen genes [8]. However, this requires advanced technology and equipment and is unsuitable for point-of-care (POC) diagnosis [9,10]. Consequently, isothermal amplification methods, such as loop-mediated isothermal amplification (LAMP), thermophilic helicase-dependent amplification (tHDA), and recombinase polymerase amplification (RPA), have the potential for POC pathogen diagnosis because they do not require thermal cyclers and are rapid, simple, and highly portable [11,12]. Among these, RPA efficiently amplifies target DNA at a constant low temperature (typically 37–42 °C) using recombinase enzymes for strand exchange and DNA polymerase [13]. RPA has the potential for efficient POC pathogen diagnosis because this is faster and simpler than other isothermal amplification technologies. Moreover, isothermal amplification reactions are often combined with visual and user-friendly platforms, such as biosensors or nucleic acid lateral flow (NALF), for simple POC diagnosis [14,15,16]. NALF uses antigen–antibody reactions to detect specific nucleic acid sequences indicative of pathogen presence [17]. This technique is facilitated through paper strip formats, enabling users to apply samples and obtain results within minutes [18].

As most foodborne pathogens are killed during food sterilization processes [19], differentiating between viable and dead bacteria can present a considerable challenge for molecular diagnostic methods [20]. Failure to accurately distinguish between the two states can produce false positives in pathogen detection, potentially triggering unnecessary recalls of food products [21]. These recalls not only incur direct costs associated with the disposal of safe products but also cause reputational damage for the food manufacturer, decreasing consumer trust, and causing subsequent economic losses [22]. Consequently, detection methods using cell membrane-permeable agents such as ethidium monoazide or propidium monoazide (PMA) have been proposed to address these issues [23]. These chemicals effectively inhibit the detection of DNA from dead bacterial cells, thereby enhancing the accuracy of rapid detection methods. A recently improved version of PMA, called PMAxx, has shown enhanced efficacy in binding to DNA from dead cells and has been applied in various isothermal amplification methods and PCR to selectively detect viable *Salmonella* species [24,25]. However, research on PMAxx-RPA-based NALF for detecting viable *Salmonella* species has not been reported.

Herein, we propose a method using PMAxx to differentiate between viable and dead *Salmonella*, applying RPA-based NALF technology for rapid *Salmonella* diagnosis, enabling fast and accurate detection. By incorporating POC diagnostics, this research offers new strategies for the detection and management of *Salmonella* in the field of food safety and health.

## 2. Materials and Methods

### 2.1. Bacterial Strains and Culture Conditions

*Salmonella enterica* subsp. *enterica* serovar Typhimurium (ATCC 14028, ATCC 43971, PT 10, PTU 302, and DT 104), *S. enterica* serovar Enteritidis (ATCC 13076), four *Salmonella* strains isolated from poultry during 2018–2019 in Republic of Korea (*S.* Enteritidis (S29), *S. enterica* serovar Paratyphi (S26), *S*. *enterica* serovar Infantis (S23), *S*. *enterica* subsp. (S13)), *Escherichia coli* (ATCC 10536), *E*. *coli* O157:H7 (ATCC 35150), *Enterococcus faecium* (KCTC 3511), *Cronobacter sakazakii* (ATCC 29004), *Pseudomonas aeruginosa* (ATCC 15692), *Bacillus cereus* (ATCC 13067), *Listeria monocytogenes* (ATCC 19117), and *Listeria innocua* (ATCC 33090) were used in this study. All bacterial strains were stored at −80 °C in tryptic soy broth (TSB; Oxoid, Basingstoke, UK) containing 50% glycerol (*v*/*v*). All strains were precultured on tryptone soy agar (TSA, Oxoid) at 37 °C overnight to obtain single colonies. Subsequently, single colonies were cultured in 10 mL of TSB at 37 °C for 16–18 h. The bacterial concentration was determined to be 10^8^–10^9^ CFU/mL by enumerating colonies on TSA plates cultured at 37 °C for 24 h. *S*. Typhimurium ATCC 14028 was used as the reference strain for optimizing PMAxx and RPA-based NALF.

### 2.2. Preparation of Viable and Dead S. Typhimurium Isolates

Cultured *S*. Typhimurium was centrifuged at 8000× *g* for 15 min at 4 °C with sterile 0.85% (*w*/*v*) NaCl (saline; Sigma-Aldrich, Taufkirchen, Germany) twice, followed by resuspension in saline, and used as viable *S*. Typhimurium. To prepare dead *S*. Typhimurium, viable *S*. Typhimurium was heated at 100 °C for 20 min and then cooled on ice for 10 min. Viable and dead *S*. Typhimurium were spread onto TSA and incubated at 37 °C for 24 h to allow colony formation. Enumeration was then performed to confirm concentrations ranging from 10^0^ to 10^9^ CFU/mL, obtained by serial dilution in sterile saline. Dead *S*. Typhimurium was also spread onto TSA plates but did not form colonies. The initial concentration of dead *S*. Typhimurium was determined to be equivalent to that of viable *S*. Typhimurium through serial dilution and enumeration in sterile saline. A pure culture was prepared containing viable *S*. Typhimurium (10^0^–10^6^ CFU/mL), dead *S*. Typhimurium (10^0^–10^6^ CFU/mL), and a mixture of viable *S*. Typhimurium (10^0^–10^6^ CFU/mL) and dead *S*. Typhimurium (10^4^ CFU/mL).

### 2.3. DNA Extraction

Bacterial genomic DNA was extracted from cell suspensions and homogenates by using PrepMan Ultra Sample Preparation Reagent (Prepman; Applied Biosystems, Foster City, CA, USA). Briefly, after collecting 1 mL of the sample in an Eppendorf tube, it was centrifuged at 16,000× *g* for 3 min to separate the supernatant. The supernatant was then removed. The pellet was resuspended in 100 μL of PrepMan, followed by incubation at 100 °C for 10 min and cooling at room temperature for 2 min. Subsequently, the mixture was centrifuged at 16,000× *g* for 3 min, and the supernatant was used as DNA. The extracted genomic DNA was stored at −20 °C until use.

### 2.4. Real-Time PCR

Real-time PCR reactions were performed using a total volume of 20 μL, comprising 10 μL of 2× SensiFAST™ Probe Hi-ROX Mix (Bioline, London, UK), 0.5 μM of forward primer, 0.5 μM of reverse primer, 0.25 μM of probe, 5 μL of DNA template, and ddH_2_O. Reactions were performed on a QuantStudio 5 Real-Time PCR System (Applied Biosystems) with initial denaturation at 95 °C for 5 min, followed by 40 cycles of denaturation at 95 °C for 10 s, and annealing/extension at 60 °C for 20 s. Results were analyzed using the QuantStudio 5 Real-Time PCR System (Applied Biosystems). Ct values >38 were considered undetectable.

### 2.5. Verification of the PMAxx Treatment

PMAxx Dye (20 mM) (Biotium, Inc., Hayward, CA, USA) was stored at −20 °C. Various concentrations of PMAxx (10, 25, 50, 75, and 100 µM) were added to viable and dead *S*. Typhimurium at a concentration of 1.34 × 10^8^ CFU/mL. After adding PMAxx, samples were incubated in the dark at room temperature for 10 min to allow penetration into damaged membranes. Samples were then exposed to a light source for 15 min at a 15-cm distance from a 500 W halogen lamp (ams-OSRAM AG, Premstaetten, Austria). Gentle mixing was performed on a shaker (Multi Shaker-FMS3-FINEPCR; FINEPCR, Gunpo-si, Republic of Korea) on ice to prevent heat damage. To remove residual PMAxx, each sample was centrifuged at 16,000× *g* for 2 min. Subsequently, DNA extraction was performed, followed by real-time PCR evaluation of interference suppression of dead cells to quantify changes in Ct and differences in Ct (dCt) induced by PMAxx. dCt was calculated by subtracting the Ct of untreated samples from that of PMAxx-treated samples as follows:

dCt = Ct _PMAxx treated_ − Ct _PMAxx untreated_

Viable and dead *S*. Typhimurium at 1.11 × 10^8^ CFU/mL were treated with the optimized PMAxx conditions. Subsequently, 200 μL of each solution was transferred to wells in a black 96-well plate (SPL, Pocheon-si, Republic of Korea), and the fluorescence intensity emitted was measured using a microplate reader (Varioskan LUX Multimode Microplate Reader, Thermo Fisher Scientific, Waltham, MA, USA). An emission spectrum ranging from 500 to 800 nm was recorded at an excitation wavelength of 450 nm. The fluorescence intensity was then measured at excitation and emission wavelengths of 510 and 610 nm, respectively. Saline (200 μL) was used as the blank.

### 2.6. RPA for NALF Detection

#### 2.6.1. Sequence Design and Synthesis

All primers and probes used in this study were synthesized and purified by Integrated DNA Technologies (Coralville, IA, USA) (Table 1). Five sets of RPA primers targeting the *invA* gene of *Salmonella* species (GenBank accession number M90846.1) were designed using Primer3Plus (www.primer3plus.com, accessed on 27 March 2023) according to the TwistDx^TM^ (Cambridge, England) guidelines. Primer sets were designed with a GC content of 40–60% to minimize dimer formation, and each primer had a 30–32 bp length. To confirm primer specificity, each primer sequence was checked against the GenBank database using BLAST (https://blast.ncbi.nlm.nih.gov/Blast.cgi, accessed on 27 March 2023). For detection in NALF, the reverse primer was labeled with biotin at the 5′ end, and the probe was designed to not overlap with the selected primers. The probe was labeled with 6-FAM at the 5′ ends, a quencher C3Spacer at the 3′ ends, and contained an internal dSpacer. Real-time PCR primers and probes were designed to detect *invA* as described by Daum et al. [26].

#### 2.6.2. RPA Assay and Primer Selection

The reaction was performed in a 50-μL volume using the TwistAmp™ Liquid Basic kit (TwistDx, Cambridge, UK). The reaction mixture included 25 µL of 2× reaction buffer, 5 µL of 10× basic E-mix, 0.45 mM dNTPs (Enzynomics, Daejeon, Republic of Korea), 0.42 µM of forward primer, 0.42 µM of reverse primer, 2.5 µL of 20× Core mix, 2.5 µL of magnesium acetate, 2 µL of DNA template, and ddH2O. RPA amplification was performed at 39 °C for 20 min using a thermocycler (SimpliAmp Thermal Cycler, Applied Biosystems, Loughborough, UK). To select a primer set, RPA was amplified using candidate primer pairs. Amplified RPA products were purified using the QIAquick PCR Purification Kit (Qiagen, Hilden, Germany), mixed with 6× Loading STAR solution (Dyne-bio, Sungnam, Republic of Korea), and loaded onto a 2% agarose gel. Visualization was performed using ChemiDoc XRS+ (Bio-Rad, Hercules, CA, USA).

#### 2.6.3. RPA-Based NALF

The modified RPA was performed in a final reaction volume of 50 μL with the additional inclusion of 0.12 µM probe and 0.1 unit/µL endonuclease IV, following the RPA assay described in Section 2.6.2. Unpurified RPA products were directly detected using NALF (BoreDa Biotech, Gyeonggi, Republic of Korea). A mixture of 10 µL of RPA product and 120 µL of NALF dilution buffer (BoreDa Biotech) was prepared and then loaded onto the sample pad of NALF. Subsequently, the mixture flowed from the sample pad to the detection area through a conjugate pad containing streptavidin and IgG-coated gold nanoparticles. The detection zone contained a test line (T) where FAM antibodies are immobilized and a control line (C) where anti-goat-IgG are immobilized, ultimately flowing onto the absorbent pad. Results were discerned after 5 min, with positive DNA amplification indicated by two lines (C and T line) and negative amplification by one line (C line). The analysis, along with representative images, was evaluated by measuring the relative peak area using ImageJ software version 1.53 (National Institutes of Health, Bethesda, MD, USA).

#### 2.6.4. Optimization of RPA Parameters

Parameters, including temperature (30 °C, 35 °C, 37 °C, 39 °C, and 43 °C), time (1, 5, 10, 15, 20, and 25 min), and endonuclease IV concentration (0.025, 0.05, 0.1, and 0.15 unit/µL) were optimized. One parameter was optimized, and the other two parameters were maintained. RPA products resulting from parameter optimization were evaluated via NALF as described in Section 2.6.3. The reaction was terminated by incubation at 65 °C for 10 min to prevent any potential additional reactions and to inactivate the enzymes.

### 2.7. Evaluation of PMAxx-RPA-Based NALF 

#### 2.7.1. Specificity and Sensitivity Assessment of PMAxx-RPA-Based NALF with Pure Culture

The PMAxx-RPA-based NALF assay was validated to assess sensitivity and specificity with a pure culture. The specificity of RPA-based NALF was validated using 10 *Salmonella* serovars and strains and eight non-*Salmonella* bacterial species. For PMAxx treatment, NALF based on DNA extraction was conducted as described in Section 2.3, Section 2.5 and Section 2.6.3 for the analytical performance. Reactions without the use of PMAxx were included for comparison.

#### 2.7.2. Detection of Viable *S*. Typhimurium Strains in Artificially Contaminated Foods

To evaluate the detection performance of viable *S*. Typhimurium in artificially inoculated food samples, raw chicken breast, and milk were purchased from local stores (Seoul, Republic of Korea). Cultured viable *S*. Typhimurium was harvested by centrifugation at 8000× *g* for 15 min at 4 °C and then washed twice with sterile saline to a final concentration of 1.11 × 10^9^ CFU/mL. The final pellet was diluted 10-fold and artificially inoculated into chicken breast and milk. Inoculated samples were homogenized with sterilized saline solution at a ratio of 1:10 to final concentrations of 10^0^–10^6^ CFU/mL. A 1-mL aliquot was collected for evaluation of the detection performance of RPA-based NALF. DNA extraction was performed according to Section 2.3, and the extracted DNA was then compared using RPA-based NALF and real-time PCR.

### 2.8. Statistical Analyses

All measurements were performed in triplicate, and results are presented as mean ± SD. Error bars represent the standard deviations obtained from three independent experiments. One-way analysis of variance was conducted using Duncan’s multiple comparison test with SPSS version 28.0.0.0 (SPSS Inc., Chicago, IL, USA). A two-sample *t*-test was performed to compare specific pairs of data points.

## 3. Results and Discussion

### 3.1. Effect of PMAxx Treatment on Viable and Dead S. Typhimurium Isolates

**Figure 1:**
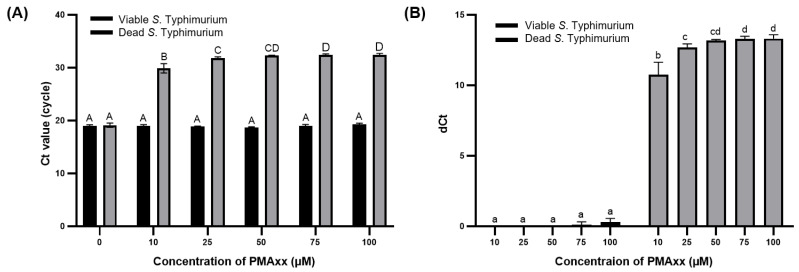
Ct values (**A**) and dCt (**B**) of DNA from viable and dead *S*. Typhimurium after PMAxx treatment at varying concentrations. Different capital letters indicate significant differences (*p* < 0.05) between the Ct values. Different lowercase letters indicate significant differences (*p* < 0.05) between dCt values.

The concentration of PMAxx was optimized to effectively suppress the amplification of DNA from dead *S*. Typhimurium and enable the selective detection of viable *S*. Typhimurium. Real-time PCR was chosen as this is a sensitive and accurate analytical method for quantifying DNA levels [27] and is suitable for evaluating the amount of DNA removed from dead *S*. Typhimurium after treatment with 0, 10, 25, 50, and 100 μM of PMAxx. The Ct values of viable and dead bacteria without PMAxx treatment did not significantly differ (*p* > 0.05). However, upon treatment with PMAxx concentrations >10 μM, the Ct values of dead *S*. Typhimurium significantly increased (*p* < 0.001) (Figure 1A). As Ct values are on a log2 scale, dCt of primer sets 1, 2, 3, 4, and 5 indicates approximately 75.000%, 87.500%, 93.750%, and 96.875% removal of bacterial DNA, respectively [28]. The dCt values for dead *S*. Typhimurium observed at PMAxx concentrations of 10, 25, 50, 75, and 100 μM of PMAxx were 10.74 ± 0.72, 12.67 ± 0.21, 13.18 ± 0.05, 13.30 ± 0.14, and 13.31 ± 0.23, respectively (Figure 1B). These results indicate the removal of 99.996%, 99.999%, 99.999%, 99.999%, and 99.999% of interference by DNA from dead *S*. Typhimurium. For viable *S*. Typhimurium, the dCt values did not significantly differ (*p* > 0.05) based on whether PMAxx treatment was applied. However, with PMAxx treatment at 75 and 100 μM, the dCt values were 0.11 ± 0.16 and 0.29 ± 0.21, respectively, indicating that approximately 51.100% and 52.060% of the viable *S*. Typhimurium DNA was affected. Gaoh et al. [29] reported that increasing concentrations of PMAxx can significantly reduce the amplification of live cell DNA. Thus, excessive PMAxx has potential cytotoxic effects on viable cells [23].

**Figure 2:**
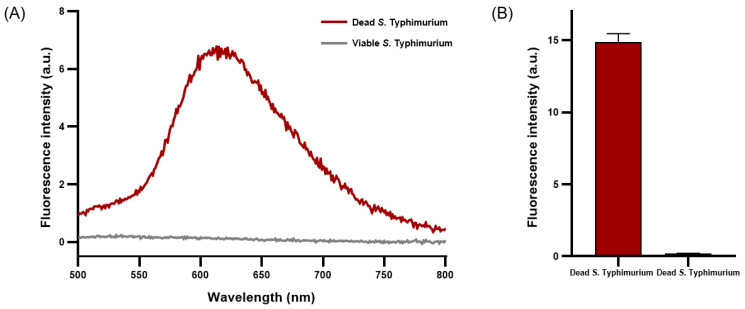
Fluorescence characteristics analysis of viable and dead *S*. Typhimurium treated with PMAxx. (**A**) Fluorescence spectrum of PMAxx-stained viable and dead *S*. Typhimurium. (**B**) Average fluorescence intensity of PMAxx-stained dead and viable *S*. Typhimurium.

PMAxx emits fluorescence near 610 nm when penetrating damaged cell membranes and crosslinking with DNA [30]. A concentration of 50 μM of PMAxx exhibited a peak emission near 610 nm, indicating maximal fluorescence intensity when applied to dead *S*. Typhimurium (Figure 2A). The average fluorescence intensity of dead *S*. Typhimurium reached 15.02 ± 0.42, whereas that of viable *S*. Typhimurium was only 0.21 ± 0.00 (Figure 2B). Under optimized conditions, a concentration of 50 μM of PMAxx was determined to be optimal for effective penetration of dead *S*. Typhimurium and crosslinking with DNA without affecting viable *S*. Typhimurium.

### 3.2. Design of Primer and Probe for S. Typhimurium Detection Using the RPA-Based NALF System

The *invA* virulence gene encodes a protein crucial for the invasion of host epithelial cells by *Salmonella* species [31]. Therefore, we designed the initial primer sets based on the coding sequence portion of *invA* to detect *S*. Typhimurium via NALF based on the RPA system. The specificity of the five primer sets was confirmed through NCBI BLAST. The expected sizes of the amplified products for primer sets 1–5 were 194, 134, 120, 162, and 188 bp. 

**Figure 3:**
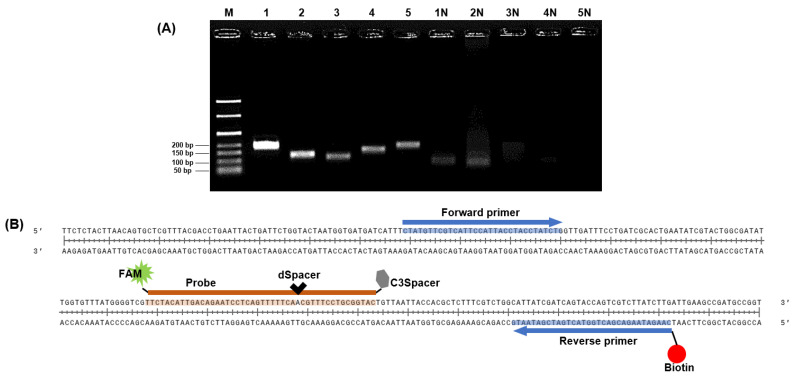
Sequence design for RPA-based NALF analysis. (**A**) Identification of predicted RPA amplification products via gel electrophoresis. Lanes M: 50 bp ladder, Lanes 1–5: Amplified positive controls using primer sets 1–5, Lanes 1N–5N: Amplified non-template controls using primer sets 1–5. (**B**) Design of probe with 5′ FAM, internal dSpacer, and 3′ C3Spacer; reverse primer labeled with biotin.

All designed primer sets effectively amplified the target gene region (Figure 3A). In gel electrophoresis, intense bands indicate a higher quantity of amplified products from the DNA template [32,33] and a successful amplification reaction. Therefore, primer set 1, which exhibited the strongest band intensity in gel electrophoresis, was selected as the primer for RPA. Typically, NALF can be easily conducted using forward and reverse primers labeled with FAM, DIG, FITC, or biotin, and several methods for detecting multiple foodborne pathogens have been reported using modified RPA primers and NALF [34,35,36]. Gel electrophoresis allows for simple discrimination of target amplicons and primer dimers based on product size differences; however, this distinction is challenging in NALF, where even small amounts of primer dimers can cause false positives [37,38]. Nevertheless, NALF is a suitable method for POC diagnosis. To prevent false positive results on NALF due to primer-dimer formation, we designed a probe complementary to the target amplicon that was labeled with FAM at the 5′ end for recognition in NALF. This probe includes a spacing region with one base replaced internally by dSpacer and an extension blocked at the 3ʹ end with a C3Spacer (Figure 3B). By adding endonuclease IV, which can cleave removed bases, to the RPA master mix, cleavage of the site where bases in the probe have been removed and extended toward the reverse primer was achieved [13]. The selected primers and probes were used in subsequent experiments.

### 3.3. Optimization of RPA Conditions for NALF-Based S. Typhimurium Detection

**Figure 4:**
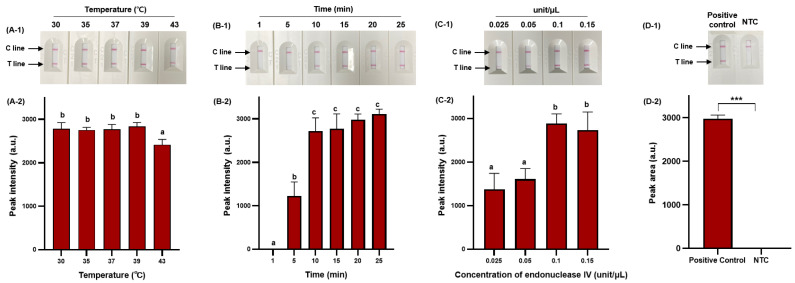
Optimization of RPA amplification conditions for NALF detection. Visualized detection of RPA-based NALF with optimization of amplification temperature (**A-1**) and relative peak intensity (**A-2**). Visualized detection of RPA-based NALF with optimization of amplification time (**B-1**) and relative peak intensity (**B-2**). Visualized detection of RPA-based NALF with optimization of endonuclease IV concentration (**C-1**) and relative peak intensity (**C-2**). Visualized detection of positive and negative RPA-based NALF under optimal conditions (**D-1**) and relative peak intensity (**D-2**). Different lowercase letters indicate significant differences in the relative peak intensity (*p* < 0.05). ***: *p* < 0.001 NTC: non-template control.

RPA offers considerable advantages in terms of low amplification temperature and rapid amplification time [39], and we, therefore, selected these as major parameters for optimization to ensure efficient RPA amplification. In addition, we optimized the concentration of endonuclease IV to ensure efficient probe cleavage. Figure 4 demonstrates the optimization process.

RPA-based NALF demonstrated amplification at all temperatures tested (30 °C, 35 °C, 37 °C, 39 °C, and 43 °C); however, a significant decrease (*p* < 0.05) in peak intensity was observed at 43 °C (Figure 4A-1,A-2). As the amplification temperature increases (>40 °C), enzyme activity may gradually decrease, and at lower temperatures, the amplification time of amplicons may exceed the rate of energy consumption, decreasing sensitivity [40,41]. Therefore, we set the optimal temperature at 39 °C to minimize the impact on amplification efficiency while ensuring effective amplification. The RPA reaction increased with amplicon quantity after 5 min, with no significant difference in peak intensity observed after 10 min (*p* > 0.05); however, the peak intensity gradually increased with extended amplification time (Figure 4B-1,B-2). Considering the variability in amplification time based on the initial template amount, a 25 min amplification time was selected to accommodate sensitive *S*. Typhimurium detection. The peak intensity of the RPA-based NALF system significantly differed based on the concentration of endonuclease IV, with maximum peak intensity at an endonuclease IV concentration of 0.1 unit/μL. A slight decrease in peak intensity was observed when 0.15 unit/μL of endonuclease IV was used and potentially attributed to miscleavage and unnecessary extension. Consequently, the optimal conditions for the RPA-based NALF system were determined to be incubation at 39 °C for 25 min with 0.1 unit/μL endonuclease IV. Positive and negative samples significantly differed (*p* < 0.001) between under optimized conditions (Figure 4D-2).

### 3.4. Validation of Detection of Viable S. Typhimurium in Pure Culture and Food Samples Using PMAxx-RPA-Based NALF 

**Figure 5:**
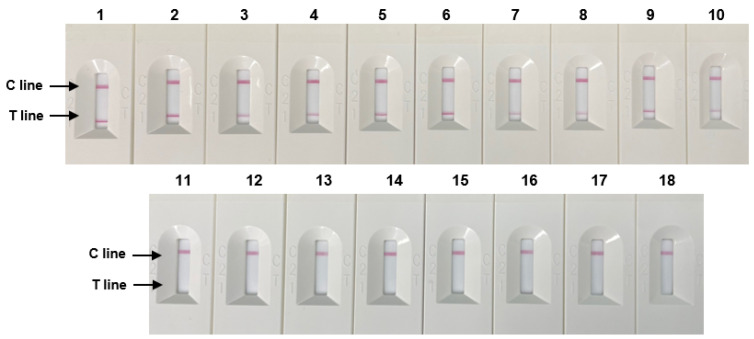
Specificity of RPA-based NALF. Lanes 1–5: S. Typhimurium (ATCC 14028, ATCC 43971, PT 10, PTU 302, and DT 104), lines 6–7: S. Enteritidis (ATCC 13076, S29), Line 8: S. Paratyphi (S26), Line 9: S. Infantis (S23), Line 10: *S. enterica* subsp. (S13), Lanes 11–18: Non-*Salmonella* species: *E. coli*, *E. coli* O157:H7, *E. faecium*, *C. sakazakii*, *P. aeruginosa*, *B. cereus*, *L. monocytogenes*, and *L. innocua*.

The specificity evaluation results of 10 *Salmonella* strains and eight non-*Salmonella* species using RPA-based NALF are shown in Figure 5. Positive results were observed on the T line of the NALF strip for *Salmonella* strains, whereas no results were detected for the T line for non-*Salmonella* species These results demonstrate the specificity of the optimized NALF based on the RPA system for *Salmonella*.

**Figure 6:**
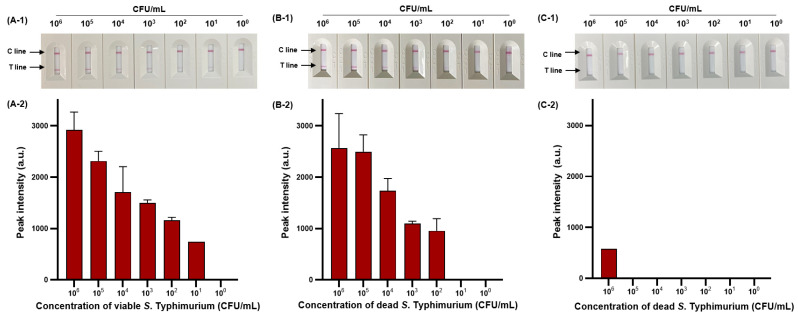
Sensitivity of RPBA-based NALF assay in pure cultures. Visualized detection of RBA-based NALF (**A-1**) and relative peak intensity (**A-2**) for viable *S*. Typhimurium. Visualized detection of RPA-based NALF (**B-1**) and relative peak intensity (**B-2**) for dead *S*. Typhimurium. Visualized detection of NALF based on RPA (**C-1**) and relative peak intensity (**C-2**) for PMAxx-treated dead *S*. Typhimurium.

**Figure 7:**
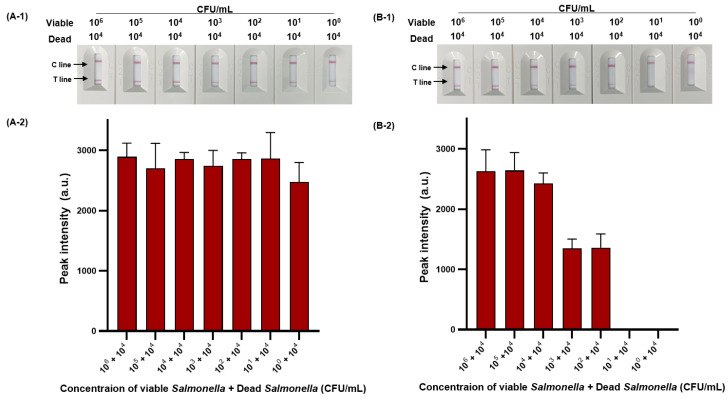
PMAxx-RPA-based NALF evaluation of viable and dead *S*. Typhimurium mixtures. Visualized detection of PMAxx-untreated RPA-based NALF with mixed viable and dead *S*. Typhimurium (**A-1**) and relative peak intensity (**A-2**). Visualized detection of PMAxx-treated RPA-based NALF with mixed viable and dead *S*. Typhimurium (**B-1**) and relative peak intensity (**B-2**).

We then evaluated the sensitivity of viable and dead *S*. Typhimurium and verified the effect of PMAxx on dead *S*. Typhimurium (Figure 6). The detection limit for viable *S*. Typhimurium was 10^2^ CFU/mL (Figure 6A-1,A-2), with only one positive result out of three replicates at a concentration of 10^1^ CFU/mL, and for dead *S*. Typhimurium was also 1.11 × 10^2^ CFU/mL (Figure 6B-1,B-2). However, after PMAxx treatment, interference from the DNA of dead *S*. Typhimurium was eliminated, producing a significant difference between the peak intensity for dead *S*. Typhimurium treated with and without PMAxx (Figure 6C-1,C-2). Furthermore, we evaluated the sensitivity by mixing dead *S*. Typhimurium at 10^4^ CFU/mL with various concentrations (10^0^–10^5^ CFU/mL) of viable *S*. Typhimurium (Figure 7). Unprocessed RPA-based NALF showed difficulty in comparing peak intensities because of interference from the dead *S*. Typhimurium DNA (Figure 7A). Conversely, after PMAxx treatment, the detection limit was 10^2^ CFU/mL (Figure 7B), similar to that for viable *S*. Typhimurium shown in Figure 6A-1,A-2, indicating the effective suppression of dead *S*. Typhimurium DNA amplification and enabling the detection of only viable *S*. Typhimurium. 

Many sterilization processes used during various processing stages do not guarantee perfect 100% sterilization [42]. The various types of bacteria in the environment do not all equally respond to the same sterilizing agent [43]. Thus, despite the efficiency of many sterilization processes, foodborne pathogens can remain in a mixed form of viable and dead cells, with the presence of dead *S*. Typhimurium in large quantities increasing the likelihood of the presence of viable bacteria proportionally [44]. Therefore, our research demonstrates that the interference removal effect of PMAxx on DNA from dead *S*. Typhimurium is sufficient.

### 3.5. Evaluation of Viable S. Typhimurium Detection in Artificially Contaminated Food Samples

**Figure 8:**
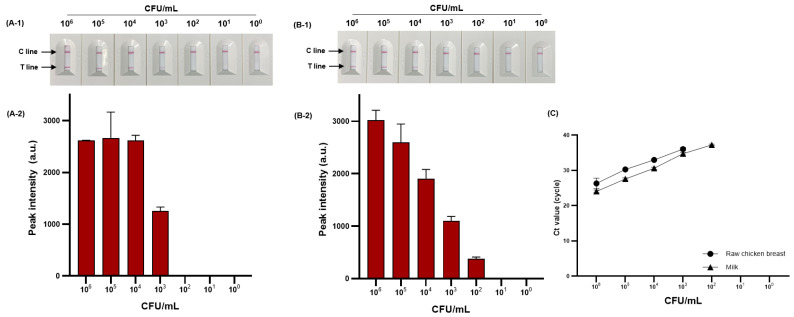
Sensitivity of RPA-based NALF in raw chicken breast and milk. RPA-based NALF (**A-1**) and relative peak intensity (**A-2**) in raw chicken breast; RPA-based NALF (**B-1**) and relative peak intensity (**B-2**) in milk; and (**C**) Ct value of real-time PCR.

We evaluated the detection performance of PMAxx-RPA-based NALF by comparing the sensitivity in food samples. The sensitivity to viable *S*. Typhimurium (10^0^–10^6^ CFU/mL) artificially inoculated into milk and raw chicken breast is shown in Figure 8A-1,A-2,B-1,B-2. The detection limit was 4.44 × 10^3^ and 5.9 × 10^2^ CFU/mL for raw chicken breast and milk, respectively. The proposed method was compared with the sensitivity and reliability of real-time PCR as the detection standard for foodborne pathogens, confirming the same detection limit of 4.44 × 10^3^ and 5.9 × 10^2^ CFU/mL for raw chicken breast and milk, respectively (Figure 8C).

NALF based on isothermal amplification methods such as LAMP and tHDA has contributed to advancing POC diagnosis of foodborne pathogens [45,46]. However, these methods typically require 60–120 min for amplification. Conversely, RPA-based NALF can complete diagnosis within 30 min. Our research results are comparable with the performance of previously reported RPA-based NALF methods for *S*. Typhimurium diagnosis. Liu et al. [34] reported a detection limit of 1.05 × 10^2^ CFU/mL for *S*. Typhimurium using RPA-based NALF in milk, raw chicken breast, and egg. In addition, Li et al. [36] reported a detection limit of 1.29 × 10^2^ CFU/mL for *S*. Typhimurium using RPA-based NALF in tomato, cabbage, and broccoli. However, these methods focused only on viable *S*. Typhimurium and did not consider the detection of dead cells. Rani et al. [47] used PMAxx-RPA-based NALF to detect viable *E. coli* O157:H7 in milk, apple juice, and drinking water, with a detection limit of 10^2^ CFU/mL. To the best of our knowledge, no research has been reported on PMAxx-RPA-based NALF for detecting viable *S*. Typhimurium.

Food matrices can inhibit molecular amplification [48] by complicating DNA extraction, denaturing DNA or enzymes, or interfering with amplification by binding to magnesium ions. For example, various polysaccharides, proteins, and blood components in meat can inhibit DNA polymerase activity, inducing competitive binding at primer-binding sites because of the binding characteristics of negatively charged particles, such as bacterial genomic DNA [49]. In addition, high concentrations of calcium ions in milk can inhibit the amplification of nucleic acid through competitive binding to DNA polymerase magnesium ions [48]. RPA has been reported to be resistant to such inhibitors. Santiago-Felipe et al. [50] successfully detected *S*. Typhimurium by using RPA even in the presence of 15–25% milk, whereas other isothermal amplification methods were only feasible at milk concentrations of approximately 14–16%. Thus, RPA has potential as a POC detection method for foodborne pathogens.

Despite these advantages, the current POC methods, including the diagnostic method developed herein, face challenges in detecting trace amounts of foodborne pathogens. Although culture-based enrichment effectively increases bacterial concentrations and dilutes inhibitors such as food components and dead cells, the overall time required for result confirmation poses a significant challenge to POC diagnosis. Zhao et al. [51] successfully detected 4 × 10^0^ CFU/25 g of *S*. Typhimurium in lamb, raw chicken breast, and broccoli using RPA-based NALF, but an additional 8 h were required for culture-based enrichment. Therefore, POC diagnosis systems for foodborne pathogens are crucial to replacing culture-based enrichment methods with faster alternatives.

## 4. Conclusions

Our study proposes a novel method that uses PMAxx to distinguish between viable and dead cells and is coupled with RPA-based NALF technology for the rapid and accurate diagnosis of *Salmonella* species. By optimizing the PMAxx concentration, we achieved a detection limit of 1.11 × 10^2^ CFU/mL for *S*. Typhimurium in pure culture. Additionally, the detection limits for *S*. Typhimurium in raw chicken breast and milk samples were 4.44 × 10^3^ CFU/mL and 5.9 × 10^2^ CFU/mL, respectively. The developed method showed promising results in spiked raw chicken breast samples, with analysis completed within 25 min at 39 °C. Moreover, our findings highlighted the sensitivity of PMAxx-RPA-based NALF in detecting viable *S*. Typhimurium in food samples with comparable sensitivity and reliability to real-time PCR, the gold standard for foodborne pathogen detection. This approach offers the advantage of rapid and accurate detection of viable pathogens, overcoming the limitations posed by conventional methods, and enabling timely intervention in food safety. Our study provides a valuable tool for the rapid and accurate POC diagnosis of viable foodborne pathogens, offering potential applications in outbreak control and food safety management.

## Figures and Tables

**Table 1:** Primer and probe sequences targeting *invA*.

Methods	Oligoname	Sequence (5′–3′)	Reference
RPA	F-1	CTATGTTCGTCATTCCATTACCTACCTATCTG	This study
	R-1	CAAGATAAGACGACTGGTACTGATCGATAATG	
	F-2	GATATTGGTGTTTATGGGGTCGTTCTACATTG	
	R-2	CTTCAATCAAGATAAGACGACTGGTACTGATC	
	F-3	GATTGCACATAAAGATCTTGTCCTCCTTAC	
	R-3	CTATCTGCTATCTCACCGAAAGATAAAACCTC	
	F-4	CATTATCGATCAGTACCAGTCGTCTTATCTTG	
	R-4	CTGAACCTTTGGTAATAACGATAAACTGGACC	
	F-5	TAACAGGATACCTATAGTGCTGCTTTCTCTAC	
	R-5	CAATGTAGAACGACCCCATAAACACCAATATC	
NALF based on RPA	F	CTATGTTCGTCATTCCATTACCTACCTATCTG	
	R	Biotin–CAAGATAAGACGACTGGTACTGATCGATAATG	
	Probe	6–FAM-TTCTACATTGACAGAATCCTCAGTTTTTCA-/dSpacer/CGTTTCCTGCGGTAC–C3Spacer	
Real-time PCR	F	GCGTTCTGAACCTTTGGTAA	[26]
	R	CGTTCGGGCAATTCGTTA	
	Probe	6–FAM–TGGCGGTGGGTTTTGTTGTCTTCT–TAMRA	

## Data Availability

The data presented in this study are available on request from the corresponding author (Data available on request due to privacy and ethical reasons).
